# Cancer-Associated *PIK3CA* Mutations in Overgrowth Disorders

**DOI:** 10.1016/j.molmed.2018.08.003

**Published:** 2018-10

**Authors:** Ralitsa R. Madsen, Bart Vanhaesebroeck, Robert K. Semple

**Affiliations:** 1 Metabolic Research Laboratories, Wellcome Trust-MRC Institute of Metabolic Science, University of Cambridge, Cambridge CB2 0QQ, UK; 2 The National Institute for Health Research Cambridge Biomedical Research Centre, Cambridge CB2 0QQ, UK; 3 UCL Cancer Institute, Paul O’Gorman Building, University College London, London WC1E 6DD, UK; 4 Centre for Cardiovascular Sciences, Queens Medical Research Institute, University of Edinburgh, Little France Crescent, Edinburgh EH16 4TJ, UK

**Keywords:** cancer, overgrowth syndromes, PI3K, PIK3CA

## Abstract

*PIK3CA* is one of the most commonly mutated genes in solid cancers. *PIK3CA* mutations are also found in benign overgrowth syndromes, collectively known as *PIK3CA*-related overgrowth spectrum (PROS). As in cancer, *PIK3CA* mutations in PROS arise postzygotically, but unlike in cancer, these mutations arise during embryonic development, with their timing and location critically influencing the resulting disease phenotype. Recent evidence indicates that phosphoinositide 3-kinase (PI3K) pathway inhibitors undergoing trials in cancer can provide a therapy for PROS. Conversely, PROS highlights gaps in our understanding of PI3K’s role during embryogenesis and in cancer development. Here, we summarize current knowledge of PROS, evaluate challenges and strategies for disease modeling, and consider the implications of PROS as a paradigm for understanding activating *PIK3CA* mutations in human development and cancer.

## Highlights

Cancer-associated activating *PIK3CA* mutations, when occurring in isolation during early development, cause a spectrum of rare disorders characterized by asymmetric, and often severe, excessive tissue growth and malformations.

Tissues are not uniformly affected, and, surprisingly, no excess of *PIK3CA*-associated adult cancers has been described.

Evidence that low-dose, repurposed cancer therapies may offer effective precision therapy is beginning to emerge.

Mouse models driven by endogenous expression of pathogenic, mosaic *PIK3CA* alleles only partly recapitulate the disease phenotype.

Although it is critical for normal cell growth and survival, the role of *PIK3CA* in early human development is poorly characterized.

## Old Drivers in a New Context

Thirty years ago this year, in 1988, the enzyme phosphoinositide 3-kinase (PI3K) was identified as a signal transducer downstream of activated cell surface growth factor receptors [1]. Its initial identification in the context of a viral oncogene immediately implicated the PI3K pathway in cancer, and we now know that the genes encoding the p110α catalytic PI3K subunit and its negative regulator, phosphatase and tensin homolog (*PTEN*), are among the most commonly mutated in solid tumors. It has long been known that heterozygous mutations in *PTEN* are also responsible for rare, cancer-prone syndromes collectively known as **PTEN hamartoma tumor syndrome** (**PHTS**; see Glossary) [2]. It is only recently, however, that we have learned of rare, but generally benign overgrowth syndromes caused by **postzygotic** activating mutations in *PIK3CA*, the gene encoding p110α [3]. Collectively known as *PIK3CA*-related overgrowth spectrum (PROS), these disorders differ from PHTS in important respects: *PTEN* mutations in PHTS are usually found in all cells, most commonly due to **germline transmission**, while *PIK3CA* mutations in PROS occur in **mosaic** form, are disproportionately found in some tissues, and appear not to be compatible with germline transmission. A key phenotypic difference lies in the increased risk of adult PI3K-associated cancer in PHTS but not PROS. Additional phenotypic variability in PROS arises from the mosaic nature of the disease and complicates efforts to establish experimental models. Nevertheless, such models are critically needed for a better understanding of this rare disorder and for preclinical testing of targeted therapies.

Study of rare diseases often improves understanding of common disorders and of fundamental biological mechanisms. Given the critical physiological role of p110α in development and growth, and its frequent pathological hyperactivation in cancer, this is potentially true for PROS, too. Conversely, candidate cancer therapeutics targeting the PI3K pathway bring hope for much needed targeted therapies for PROS, as recently demonstrated in an uncontrolled case series treated with the p110α-specific inhibitor Alpelisib (BYL719) [4]. Increased awareness of PROS thus seems timely. This review summarizes current knowledge of p110α activation in PROS, outlines key unanswered questions, and discusses challenges and opportunities in disease modeling and evaluation of novel therapies.

## Regulation of p110α Activity

**Figure 1:**
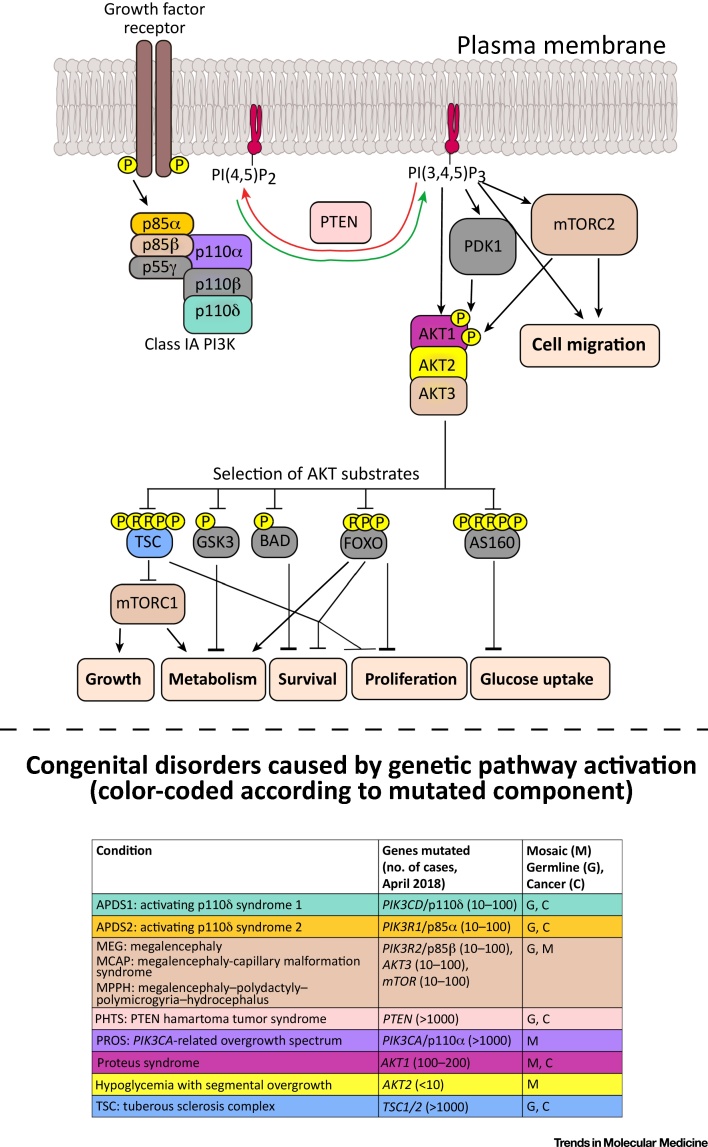
Simplified PI3K Signaling Cascade and Known Clinical Disorders Caused by Genetic Pathway Activation. Color coding corresponds to the genetic disorders listed. The numbers of reported cases in the table are approximate. Cancer (C) is used to indicate a known increase in malignancy risk for each disorder. Although cancer itself features complex genetic mosaicism, in this figure ‘mosaic’ (M) is restricted to non-cancer mosaicism. AKT, protein kinase B; FOXO, Forkhead Box O; GSK3, glycogen synthase kinase-3; mTORC, mechanistic target of rapamycin complex; PI3K, phosphoinositide 3-kinase; PI(3,4,5)P_3_, phosphatidylinositol-3,4,5-trisphosphate; PI(4,5)P_2_, phosphatidylinositol-4,5-bisphosphate; PTEN, phosphatase and tensin homolog.

The PI3K enzyme first identified was the prototype of what is now known as Class I PI3Ks, which catalyze conversion of the membrane lipid phosphatidylinositol-4,5-bisphosphate [PI(4,5)P_2_] to the second messenger PI(3,4,5)P_3_ (also known as PIP_3_). Class I PI3K catalytic subunits are divided into two subclasses – IA and IB – based on differential usage of regulatory subunits. Class IA PI3Ks are heterodimers of one of three catalytic subunits (the *PIK3CA* gene product p110α, the *PIK3CB* product p110β, or the *PIK3CD* product p110δ), tightly bound to one of five regulatory subunits (the *PIK3R1* gene products p85α/p55α/p50α, the *PIK3R2* product p85β, or the *PIK3R3* product p55γ; Figure 1). p110α and p110β are widely expressed, with p110δ predominantly found in leukocytes [5].

p110α signals downstream of plasma membrane-associated tyrosine kinases via recruitment of the p85 subunit to tyrosine-phosphorylated receptors/receptor-associated adaptor proteins, or by direct binding to the RAS oncogene [5]. p110α activation leads to acute increases in PIP_3_ and its degradation product PI(3,4)P_2_, stimulating membrane recruitment of effector proteins with PIP_3_/PI(3,4)P_2_-binding domains such as the pleckstrin homology domain. Protein kinase B (AKT) serine/threonine kinases are the best studied PI3K effectors, regulating cell growth, metabolism, survival, and proliferation 5, 6.

PI3K activity is tightly constrained, resulting in transient and localized PIP_3_ generation. Both negative feedback by downstream pathway components and the activity of several phospholipid phosphatases are involved in signal termination. Most important of the known lipid phosphatases is the tumor suppressor PTEN, which converts PIP_3_ back to PI(4,5)P_2_ [5]_._ The importance of exquisite regulation of PI3K signaling is exemplified by the growing number of genetic disorders known to be caused by mutations in pathway components (Figure 1).

## *PIK3CA* Mutations in Cancer

PI3K activity was linked to pathological cell growth and oncogenesis early after its discovery, but it was not until 2004 that somatic mutations in *PIK3CA* were reported in cancers [7]. Through high-throughput sequencing, genetic hyperactivation of PI3K/AKT signaling has now become recognized as one of the most frequent ‘driver’ mechanisms in many cancers [5]. Pan-cancer analyses by The Cancer Genome Atlas identified *PIK3CA* and *PTEN* among the genes most frequently harboring somatic point mutations in more than 12 different solid tumor types, only behind the tumor suppressor gene *TP53* 8, 9. Cancers with a high prevalence of activating *PIK3CA* mutations include breast (>30%), endometrial (>30%), bladder (>20%), colorectal **carcinoma** (>17%), and head and neck squamous cell carcinoma (>15%) 8, 9, 10, 11.

**Figure 2:**
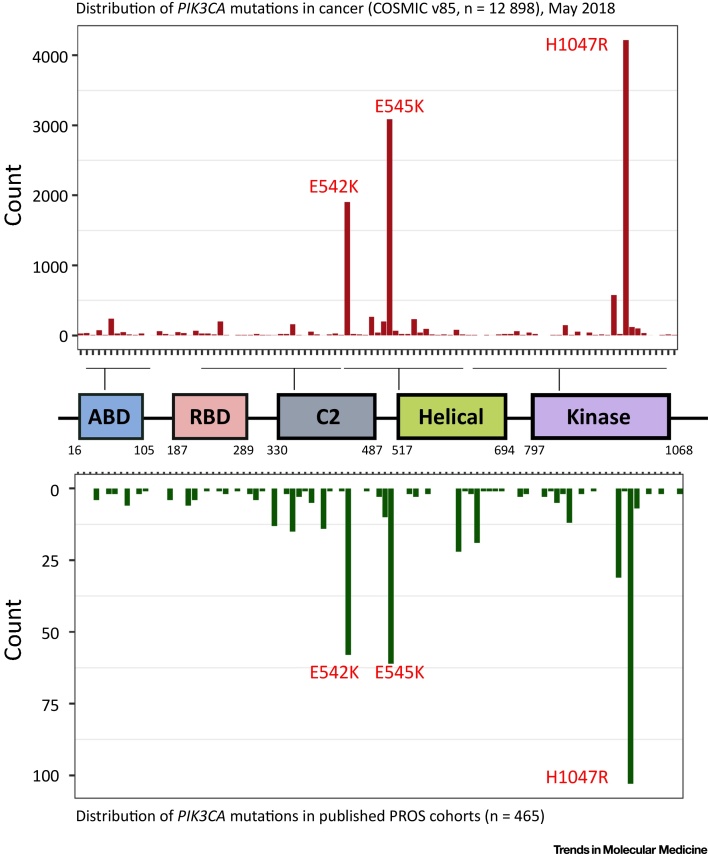
The Spectra of Activating *PIK3CA* Mutations in Cancer and PROS. Most (>80%) activating *PIK3CA* mutations in cancer (top) and PROS (bottom) cluster at three hotspots: two glutamic acid (E) residues at codons 542 and 545, and a histidine (H) residue at codon 1047. The most frequent mutations at these sites introduce a strongly positively charged side chain – lysine (K) at codons 542 and 545 or arginine (R) at codon 1047. With the exception of the RAS-binding domain (RBD), mutations affect the entire p110α protein, including the adaptor-binding domain (ABD), the C2 domain, the helical domain, and the kinase domain. These domains are all required for the inhibitory interactions between p110α and its regulatory subunit. Moreover, the C2 and kinase domains act as lipid-binding interfaces. The distribution of *PIK3CA* mutations in cancer were obtained from The Catalogue of Somatic Mutations in Cancer (COSMIC, v85, May 2018) [63], filtered for variants with ≥10 counts (except if also found in PROS). *PIK3CA* mutations in PROS comprise published cases from larger cohort studies 17, 22, 64, 65, 66, 67. Note that the *x*-axis is not numerical and thus does not scale to the distance between the indicated residues. PROS, *PIK3CA*-related overgrowth spectrum.

Cancers may also have gene amplification or overexpression of any p110 isoform, but only p110α is commonly mutationally activated [5]. Mutations span the entire p110α protein except the RAS-binding domain. Over 80% of *PIK3CA* mutations cluster at three sites, or ‘hotspots’, namely, glutamates (E) 542 and 545 in the helical domain, and histidine (H) 1047 near the C terminus of the kinase domain (Figure 2). These hotspot variants have the most potent effect on enzymatic activation and downstream biological responses (Table S1 and 11, 12).

## *PIK3CA* Mutations in Benign Overgrowth

Although established as cancer drivers, *PIK3CA* hotspot mutations were also, surprisingly, identified in benign skin lesions known as **epidermal nevi** and **seborrheic keratoses** [13]. As in cancer, mutations were only found in the lesions, thus representing another example of genetic mosaicism. More recently, postzygotic, mosaic, activating *PIK3CA* mutations were also identified in several different forms of segmental overgrowth – that is, asymmetric overgrowth affecting only some parts of the body 14, 15, 16. Since then, a wide spectrum of such conditions has been attributed to mosaic genetic activation of p110α. Many affected patients have patterns of overgrowth previously labeled as specific syndromes. The resulting fragmented and inconsistently applied nomenclature complicates classification and fails in the face of intermediate syndromes, leading to the proposed designation of a ‘*PIK3CA*-related overgrowth spectrum’, or PROS, to capture the disorders under one label and to reflect disease etiology [3].

### Clinical Features of PROS

**Figure 3:**
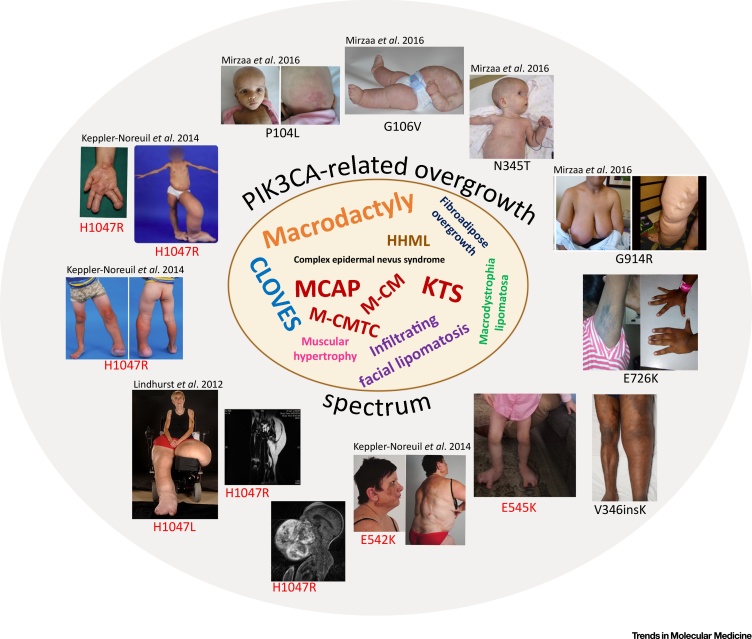
PROS and Some of Its Constituent Disorders. Many overgrowth disorders previously classified on the basis of anatomical differences are now known to share a common genetic etiology, namely, postzygotic mosaic, activating mutations in *PIK3CA*. A selection of older descriptors, many of persisting, practical clinical value, is shown in the central word cloud, with approximate frequency of use of the terms indicated by font size. PROS is a more general term proposed to reflect the common genetic etiology of these conditions. Representative images capturing different manifestations of PROS are shown, with the causal *PIK3CA* mutation in each case indicated below the image. Radiologic images are sections of magnetic resonance images illustrating asymmetric fatty leg overgrowth and infiltrating facial lipomatosis. Hotspot mutations are shown in red. Where people in images are identifiable, informed consent has been gained for publication. References are given for images obtained from published reports 3, 14, 17. CLOVES, congenital lipomatous overgrowth, vascular malformations, epidermal nevi, scoliosis syndrome; HHML, hemihypertrophy with multiple lipomatosis; KTS, Klippel–Trenaunay syndrome; MCAP, megalencephaly-capillary malformation syndrome; M-CM, macrocephaly-capillary malformation syndrome; M-CMTC, megalencephaly-cutis marmorata telangiectatica congenita; PROS, *PIK3CA*-related overgrowth spectrum.

The severity of PROS is highly variable, ranging from localized overgrowth, for example of a digit, to severe, extensive, and life-threatening overgrowth affecting major vessels and/or critical organs (Figure 3). PROS may be conceived of as a highly anatomically variable admixture of overgrown tissues, with vasculature (capillaries, veins and lymphatics) and adipose tissue often most dramatically affected macroscopically. Many other tissues and organs, including bone, brain, peripheral nerves, liver, skeletal and cardiac muscle, may also be affected 3, 17, 18. Overgrowth manifests at birth, and progressive overgrowth during childhood is the norm. Soft-tissue overgrowth sometimes persists in adult life, but this cannot currently be predicted. Few reports have identified the identity of mutated cells, with genotyping usually performed on whole tissue. However, *PIK3CA* mutations have been detected in subcultured dermal fibroblasts [14], adipocytes [16], lymphatic endothelial cells 19, 20, and skin (epithelial) keratinocytes [21]. Hotspot *PIK3CA* mutations are only very rarely identified in lymphocyte DNA, even when the overall disease burden is extensive 17, 22.

Available therapy for PROS centers on judicious surgical ‘debulking’ of affected regions, with procedures often hazardous due to abnormal vascular anatomy. Surgical or radiological blocking of overgrown blood vessels is also important. Current clinical practice and access of patients to services show major geographical variation, and the unmet need for targeted, less disfiguring approaches to therapy is large.

### Mutational Spectrum and Genotype–Phenotype Correlation

**Figure 4:**
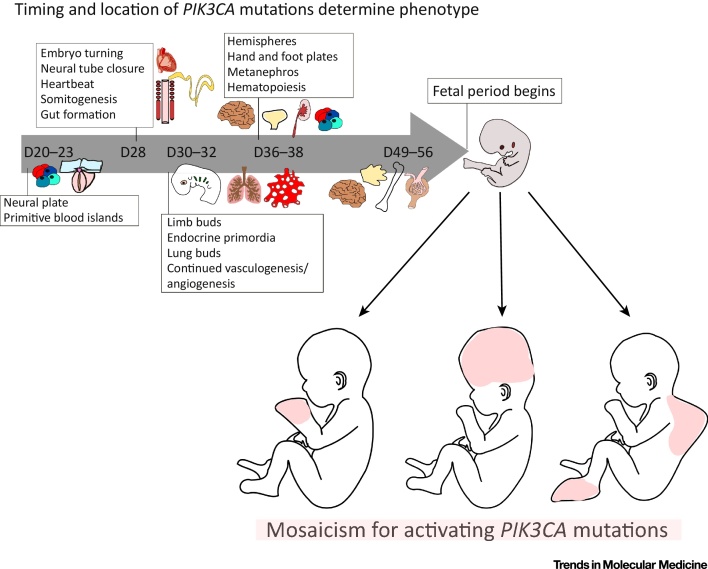
Key Figure: The Phenotypic Effect of Developmental Timing and Location of Founder *PIK3CA* Mutations

The severity of PROS is most dependent on the timing and location of the initiating mutation (Figure 4, Key Figure). The profile of *PIK3CA* mutations in PROS closely resembles that in cancer (Figure 2), and hotspot mutations have been suggested to be associated with more severe, focal overgrowth, with rarer non-hotspot mutations often causing more widely distributed but milder overgrowth 3, 17, 22. However, this fails to explain the full range of observed phenotypes, with clinical observations and biochemical studies of non-hotspot mutations suggesting a more graded phenotypic spectrum 12, 17, 22. The variability arising from different anatomical distributions of mutations means that very large studies will be required to address a possible genotype–phenotype correlation more definitively.

## Unanswered Questions about PROS and Activating *PIK3CA* Mutations

### Is PROS Explained by Cell-Autonomous Consequences of PI3K Activation?

Most studies of cancer and PROS have concentrated on cell-autonomous effects of p110α activation on processes such as cell growth, survival, and migration. However, the mutation burden in PROS at sites of overgrowth is commonly less than the 50% expected if all cells in the tissue were heterozygous for the mutation, and overgrown tissue contains multiple cell types of different embryonic origin. This raises the possibility, still to be tested, that *PIK3CA* mutation-positive cells exert growth-promoting effects on adjacent or distant cells. This could involve cell–cell interactions, paracrine growth factors, **exosomes**, and/or alterations to the extracellular matrix.

### What Explains the Tissue-Selective Overgrowth of PROS?

**Figure 5:**
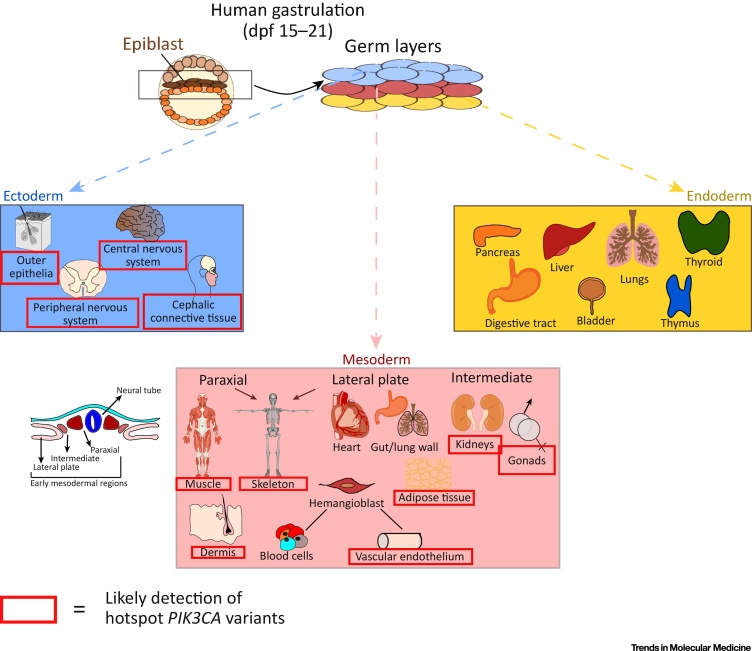
Apparent Skewing of Hotspot *PIK3CA* Mutations toward Some Embryonic Lineages. Shortly after fertilization, the human embryo resembles a flattened cellular disk known as the epiblast. Between 15 and 21 days postfertilization (dpf), the epiblast transforms into the three germ layers (ectoderm, mesoderm, and endoderm) in the process of gastrulation. This involves carefully balanced morphogen gradients and intricate crosstalk among the developing germ layers, acting on one another to instruct lineage specification. The ectoderm gives rise to the epidermis (skin) and nervous system (neuroectoderm), including neural crest derivatives (e.g., cephalic connective tissue). The mesoderm is patterned into three subtypes (shown in the transverse section of the developing embryo), which combined develop into a variety of tissues, including bone, cartilage, connective and adipose tissue, smooth and skeletal muscle, the vascular system, heart, gut and lung walls, blood cells, kidneys, and gonads. The endoderm lineage specifies the gastrointestinal and respiratory systems, the epithelial lining of the bladder and urethra, as well as many endocrine glands. In PROS, it appears that hotspot *PIK3CA* mutations such as H1047R are not present in endodermal tissue derivatives, and further exhibit a skewing away from blood cells. Note that biopsies from internal organs are rarely available for direct genotyping, and inferences about mutation distribution in such cases are largely based on the macroscopic pattern of overgrowth. PROS, *PIK3CA*-related overgrowth spectrum.

The initial mutation driving PROS is assumed to arise stochastically, and therefore the probability of different developmental lineages being affected should reflect the number of cells of each lineage present in the embryo at the time of mutation. PROS, however, exhibits apparent skewing in the pattern of overgrowth among tissues, with mesoderm-derived tissues (e.g., adipose tissue, vasculature, muscle, bone) and **neuroectoderm**-derived tissues (e.g., brain, cephalic connective tissue) prominently affected macroscopically (Figure 5). There is much less macroscopic involvement of endoderm-derived structures (e.g., pancreas, liver), and little evidence of epithelial overgrowth beyond epidermal nevi and seborrheic keratoses, both of neuroectodermal origin [23]. The extremely low burden of *PIK3CA* hotspot mutations in blood, in contrast to non-hotspot variants, which are not infrequently detected in many tissues, including blood, is also of note 15, 17, 18, 22. These observations could be accounted for by processes such as skewing of developmental cell fate decisions, lineage-specific positive or negative selection for *PIK3CA*-mutant cells, and/or alterations of stem cell dynamics. For instance, hotspot *PIK3CA* variants may lead to lineage-specific cell loss during or after differentiation due to mechanisms such as **oncogene-induced senescence**, which is known to occur in cells with strong activation of PI3K signaling [24]. Hyperactivation of PI3K signaling in stem cells may also lead to attenuation or ‘exhaustion’ of stem cells’ regenerative capacity, a phenomenon best studied in hematopoietic stem cells [25].

Increased PI3K signaling may affect **stemness** and/or early lineage determination of pluripotent stem cells (PSCs), and even subtle effects could have a major influence on the PROS phenotype. PI3K/AKT signaling contributes to **self-renewal** and stemness in models of early development 26, 27, but understanding of dose-dependent effects on human cell fate decisions and crosstalk with key ‘stemness’ pathways is sparse. The development of human stem cell-based models with activating *PIK3CA* mutations will permit interrogation of the apparent lineage skewing observed *in vivo*. The potential role of neural crest stem cells in PROS will be of particular interest given their ability to generate neuroectodermal and mesodermal tissue derivatives, corresponding to tissues most commonly overgrown in PROS.

It is important to stress that most diagnostic testing is currently undertaken on accessible areas of macroscopic overgrowth rather than on tissues from internal organs, especially if not frankly enlarged, creating an inevitable bias. Apparent tissue-selective overgrowth may also partly reflect nonuniform *PIK3CA* gene expression, with the pancreas and liver having more than five times lower mRNA expression than arteries, nerves, adipose tissue, uterus, and breast [28]. Moreover, tissues also differ in their capacity to expand as part of physiological adaptation. Adipose tissue and vessels, for example, may dramatically grow and later regress in the face of a transient positive energy balance or tissue injury, and this inherent plasticity may be amplified into greater overgrowth by an endogenous trophic stimulus. More systematic tissue sampling will be needed, ideally with single-cell sequencing, to form an unbiased view of *PIK3CA* mutation distribution in PROS.

### Why Is Cancer Not More Common in PROS?

To date, the only malignancy reported in PROS is Wilms tumor (nephroblastoma), an embryonal pediatric cancer identified in four of around 200 patients with PROS [29]. Thus, although *PIK3CA* mutations are very common in cancers, none of the cancer types enriched for such mutations (e.g., endometrial and breast) have been reported in PROS. This may reflect distinct mutation tissue distributions in PROS and cancer. Indeed, as mentioned earlier, most macroscopic overgrowth in PROS occurs in mesodermal and neuroectodermal derivatives (Figure 5), while *PIK3CA*-associated cancers most commonly arise in ectodermal or endodermal epithelia. Neuroectodermal and mesodermal cancers show very low occurrence, if any, of activating *PIK3CA* mutations [10].

It is also important to emphasize that when denoting a cancer-associated mutation, a cancer ‘driver’ implies that it confers a selective growth advantage at some point during cancer development, and not that it is sufficient for cancer initiation or maintenance [30]. This is illustrated by murine cancer models which demonstrate that activating *Pik3ca* mutations usually require cooperating genetic lesions to induce cancer 31, 32, 33. The cellular/tissue context of the mutations is also likely to be an important determinant of their effect on cell behavior, with evidence that even within a single tissue such as the mammary gland, the effect of the hotspot variant *Pik3ca*-H1047R is dictated by the cell of origin 33, 34.

### Why Does PTEN Deficiency Not Resemble PROS in Cancer Risk?

In marked contrast to PROS, PHTS predisposes to several cancers associated with PI3K activation [35], including breast, endometrial, and colorectal carcinomas. This may reflect expression of the *PTEN* mutation in all cells, or the fact that PTEN is not specific for p110α-derived PIP_3_. Instead, it opposes signaling by any Class I PI3K isoform. Moreover, PTEN has nuclear and lipid phosphatase-independent activities 36, 37, although their role in the PHTS phenotype is unclear. A further interesting possibility is that different profiles or strengths of aberrant PI3K activation influence cancer risk. Side-by-side comparisons have not been published, but baseline pathway activation by *PIK3CA* mutations appears higher than that conferred by heterozygous *PTEN* loss of function, which results in only modestly increased basal PI3K signaling in some contexts 38, 39, and no discernibly increased signaling in others 39, 40. Dose-dependent effects of PI3K activation have also been documented in the myriad of cellular studies comparing *PIK3CA* mutations of different potency (see Table S1 and 11, 12), as well as in mouse models of *PIK3CA*-driven cancer where tumorigenesis is often only observed upon transgenic overexpression of mutant *PIK3CA* 32, 33, 34, 41, 42, 43, 44, 45, 46, 47, 48, 49. Thus, understanding the mechanisms determining differential cancer risk in PHTS and PROS may yield new biological insights into Class IA PI3K and will be important in guiding personalized therapy.

### Will PI3K Pathway Inhibition Be Effective in PROS?

Downstream inhibition of the PI3K pathway by targeting of mechanistic target of rapamycin (mTOR) is currently possible with agents such as Sirolimus, already used in clinical practice for prevention of organ allograft rejection, for the treatment of autoimmune disorders and some cancers, and, more recently, for precision therapy for tuberous sclerosis complex [50]. Published and unpublished cases suggest that sirolimus may exert some beneficial effects in PROS, and its use on compassionate grounds is consequently spreading in clinical practice. Nevertheless, the lack of randomization, blinding and placebo control in current studies means that its true efficacy is currently difficult to assess.

A more targeted option for therapy would be specific inhibition of p110α. Several small-molecule inhibitors of p110α are in development for oncology indications (Table S2), but have been ineffective as monotherapy in cancer. One problem has been drug intolerance, especially at the high doses routinely used in cancer trials. Nevertheless, the palette of available PI3K inhibitors can potentially be repurposed for treatment of PROS, where low-dose single-agent therapy may suffice. While no p110α-specific inhibitors have yet been approved for clinical use, one promising option is the ATP-competitive p110α inhibitor Alpelisib (BYL719; Novartis). A low dose of Alpelisib used on compassionate grounds was shown in an uncontrolled case series to have striking effects in a cohort of 19 PROS patients, several with life-threatening complications [4]. The low dose of Alpelisib provoked few side effects and did not impair linear growth of the often young patients [4]. This early indication of possible efficacy with good tolerability urgently requires assessment in formal trials. It is very encouraging evidence that a therapeutic window may be found, permitting the needed lifelong treatment which is likely to be growth suppressive rather than curative [51]. Previous studies have shown that insulin/PI3K/mTOR inhibition prolongs lifespan across a range of evolutionarily distinct organisms [52], and low-dose p110α inhibition in obese mice and monkeys increases energy expenditure and reduces adiposity, with long-term metabolic benefits 53, 54. These observations suggest that there may even be collateral benefits of low-dose PI3K inhibition beyond mitigating disease-specific features of PROS.

## Models of *PIK3CA* Activation and PROS: Challenges and Opportunities

Faithful experimental models of PROS, ideally spanning a spectrum of mutations, are required to obtain mechanistic insight into the underlying disease pathology and to test proposed therapeutic regimens. Several approaches to PROS modeling in mice have been described (Table 1), and these illustrate the complexity of modeling mosaic disease, arising from the variability in time and place of the initiating mutation. Important decisions to be made in PROS modeling include (i) whether to employ global versus tissue- or lineage-restricted *Pik3ca* mutation; (ii) which *Pik3ca* allele to use; (iii) whether to activate *Pik3ca* in all cells in the tissue studied or instead to use subthreshold induction to yield mosaic activation; and, critically (iv) which time window to use for induction.

**Table 1:** Activating *Pik3ca* Mutations in Mouse Models of Relevance to PROS with Information about Expression Strategy and a Summary of Key Findings^a^

Mouse model	Expression	Time of induction	Lineage/tissue specificity	Pathology	Refs
*Cre-del; Pik3ca^WT/H1047R^* *hACTB-Flpe; Pik3ca^WT/H1047R^*	Endogenous	Congenital	Ubiquitous	Embryonic lethality (E9.5–E10.5) with failed turning, absence of several somites, failed neural tube closure, vascular defects, and impaired hematopoiesis.	[55] [42]
*T-CreER; Pik3ca^WT/H1047R^* (MosMes-*Pik3ca^H1047R^)*	Endogenous	E7.5–E10.5	Mesoderm	Subcutaneous vascular malformations at birth at multiple sites. Impaired survival upon strong induction. No somatic overgrowth.	[57]
*hGFAP-Cre; Rosa26-rtTA; TetO-PIK3CA^H1047R^*	Transgenic	Congenital or P1	Subset of neural progenitors (telencephalon from E13.5)	Congenital expression led to brain malformations and death of pups before weaning. Postnatal induction did not induce anatomical brain abnormalities.	[58]
*hGFAP-Cre; Pik3ca^WT/E545K^*^b^	Endogenous	Congenital	Subset of neural progenitors (telencephalon from E13.5)	Viable with large dysplastic brains but no gross brain malformations.	[58]
*Nestin-Cre; Pik3ca^WT/E545K^*^b^ *Nestin-CreER; Pik3ca^WT/E545K^*^b^	Endogenous	Congenital or from P1	Neural progenitors (telencephalon from E11)	Embryonic induction increased brain size with dysplasia. Postnatal induction produced no anatomical brain abnormalities but did cause epilepsy which was reversible by pan-PI3K inhibition.	[58]
*Tie2-Cre; Pik3ca^WT/H1047R^* *Tie2-Cre; Rosa26-Pik3ca^H1047R^*	Endogenous or transgenic	Congenital	Endothelial cells	Embryonic lethality (E10–E11.5) due to vascular defects and impaired hematopoiesis. Linear growth retarded. Growth and vascular phenotype rescued by p110α-specific inhibitor administered at E7.5–E9.5.	[55] [59] [62]
*Pdgfb-CreER; Pik3ca^WT/H1047R^*	Endogenous	P1	Endothelial cells	Retinal endothelial cell hyperplasia, absent pericytes, and loss of arteriovenous identity markers at P6. Reversed by mTOR inhibition with rapamycin.	[57]
*Cdh5-CreER; Rosa26-Pik3ca^H1047R^*	Transgenic	8–10 weeks old	Endothelial cells	Mortality within 15 days of induction with cardiac degeneration. Intramuscular expression caused bleeding from vascular malformations, and increased vessel density. Reduced with either dual PI3K/mTOR or mTOR inhibition.	[62]
*CAG-CreER; Rosa26-Pik3ca^H1047R^* *UBC-CreER; Rosa26-Pik3ca^H1047R^*	Transgenic	3 weeks old	Ubiquitous	Venous malformations with endothelial cell hyperplasia and death within 50 days of induction. Transplanted malformations responsive to p110α or mTOR inhibition.	[59]
*UBC-CreER; Pik3ca^WT/H1047R^* *CAG-Flpe-ER; Pik3ca^WT/H1047R^*^c^	Endogenous	6–8 weeks and 15 weeks old [60] 8 weeks old [42]	Ubiquitous	Hypoglycemia, hypoinsulinemia, organomegaly, and increased vascularization within 3 weeks of induction; death within 100 days [60]. Sudden death for unknown reasons (median survival 220 days after induction) [42].	[60] [42]

a Abbreviations: 4-OHT, 4-hydroxytamoxifen; Cdh5, Cadherin 5; Cre-del, Cre deleter; ER, estrogen receptor; Flpe, flippase; hACTB, human β-actin; hGFAP, human glial fibrillary acidic protein; MosMes, mosaic mesoderm; P#, postnatal day; Pdgfb, platelet-derived growth factor β; rtTA, reverse transcription activator (‘tet-on’ system); T, T Brachyury; TetO, bacterial tet operator site; Tie2, tunica intima endothelial kinase 2; UBC, ubiquitin.

b *Pik3ca* expression from targeted allele disrupted in cells without Cre expression.

c Hypomorph with reduced *Pik3ca* expression until *neo* cassette removal.

Mice with constitutively expressed *Pik3ca*-H1047R from the endogenous locus die at E9.5–E10.5 42, 55, consistent with Happle’s hypothesis that many mosaic disease variants would be lethal in the germline [56]. It is plausible that some of the less common *PIK3CA* alleles are compatible with embryonic survival, given their detection in many tissues in PROS patients, but this has not yet been assessed. To overcome early lethality and to mimic PROS, varying levels of *Pik3ca*-H1047R mosaicism in mesoderm have been generated using inducible **CreER** expression driven by the *T Brachyury* promoter [57]. Mutant animals developed vascular abnormalities remarkably similar to human venous malformations, with severity relating to mutation burden, but surprisingly no other tissue overgrowth was seen. It is not clear whether this reflects biological differences between mice and humans, the promoter used, or the timing of mutation induction.

The importance of mutation timing and cellular context has been shown in studies modeling *Pik3ca*-driven brain overgrowth in mice, using Cre-dependent transgenic *PIK3CA*-H1047R or endogenous *Pik3ca*-E545K expression [58]. These studies demonstrated that brain overgrowth requires p110α activation during embryogenesis, while postnatal induction of *Pik3ca*-E545K is sufficient to cause neurological dysfunction in animals with normal brain size [58]. Other mouse models featuring inducible whole-body *Pik3ca*-H1047R expression have yielded somewhat contradictory results, whether expressing the mutant allele transgenically [59] or from the endogenous *Pik3ca* locus 42, 60 (Table 1). The timing and strength of mutant induction have emerged as key variables affecting disease severity, but do not fully explain the observed differences. A recently developed model utilizing an inducible, artificially activated p110α variant did develop the majority of phenotypes observed in PROS [4], despite postnatal induction. The variant used confers stronger pathway activation than natural hotspot *PIK3CA* mutants, suggesting that mice differ from humans in requiring a higher strength of aberrant PI3K signaling to express the full somatic growth phenotype of PROS [4].

### Emerging Opportunities for PROS Modeling

Aberrant growth in PROS begins before birth, so study of mosaic embryonic p110α activation is important. Given species differences in early development, interrogation of developmental functions of p110α in human models is highly desirable. Recent methodological advances in stem cell science offer many new experimental options. Reprogramming of a mosaic culture of cells from patients with PROS to **induced PSCs (iPSCs)** may yield an initial admixture of mutant and wild-type cells, from which **isogenic** clonal wild-type and mutant iPSCs may simultaneously be established. Alternatively, **clustered regularly interspaced short palindromic repeats (CRISPR)-based gene editing** can be used to engineer normal human PSCs with different activating *PIK3CA* mutations, generating an allelic series on an isogenic background. In turn, these cells can be used to obtain 3D or 4D ‘multiorgan’ systems, bridging the gap between 2D cellular studies and animal-based models [61]. Mosaicism can also be modeled in a controlled manner by mixing wild-type and mutant human PSCs, which could be labeled to permit visualization of population dynamics during differentiation *in vitro* or *in vivo*. Inducible systems will further allow individual *PIK3CA* mutations to be expressed at different time points and can be used to address questions about negative selection during lineage specification. Importantly, PSCs can be used to generate all the cell types affected in PROS, enabling studies of cell-specific pathology as well as preclinical drug testing.

## Concluding Remarks

The discovery of activating *PIK3CA* mutations in PROS has not only yielded a new target for much-needed precision therapies, but has also exposed gaps in our understanding of PI3K signaling in human cancer and embryonic development (see Outstanding Questions and Box 1). A better understanding of how PI3K pathway responses differ depending on signaling dosage and cellular context may even suggest novel therapeutic strategies in PI3K-driven cancers. Monotherapy with high-dose PI3K-specific inhibitors has fared poorly in oncology trials, and strategies in which PI3K inhibition is more closely tailored to the underlying perturbation may be worthy of assessment [51]. Key insights are expected from single-cell studies of human PROS, and from comparison of spatiotemporal dynamics of PI3K signaling in different PI3K-related genetic diseases. Temporally controlled cell type-specific expression of mutant *PIK3CA* alleles in animal models, allied to creative use of engineered human stem cells and organoids, is likely to be an important tool. Such efforts will require cross-discipline collaborations including basic and translational scientists focusing on signaling, cancer, and development.

Outstanding Questions **Is PROS adequately explained by cell-autonomous actions of *PIK3CA* mutations?** Clinical observations in PROS suggest circumstantially that *PIK3CA* mutant cells might exert paracrine or remote effects on unaffected cells, with consequences for disease outcome. This remains to be substantiated, but could involve signal propagation through secreted biomolecules or exosomes.

**What explains the tissue-selective overgrowth of PROS?** The apparent sparing of endoderm and hematopoietic lineages suggests negative as well as positive selection acting on cells with mutations. Approaches such as stem cell-based modeling using admixtures of mutant and wild-type cells, and single-cell sequencing to reconstruct cell lineages *in vivo* offer great potential for better understanding of the cellular ecology of PROS.

**Why does PROS not confer a greater risk of known *PIK3CA*-associated cancers?** To date, PROS patients have not been reported to exhibit any increased risk of *PIK3CA*-associated adult cancers. This might be explained by differences in the tissues and cell lineages that are affected, or may reflect the importance of cooperating mutations for cancer development. Better understanding of context-dependent consequences of chronic *PIK3CA* activation could offer fundamental insights into the pathogenesis of *PIK3CA*-associated cancers.

**What explains the different clinical consequences of *PTEN* loss and *PIK3CA* activation?** They may be explained by p110α-independent PI3K activity, by loss of non-lipid phosphatase roles of PTEN, or by differences in the pattern of deranged PI3K signaling resulting from *PTEN* loss and *PIK3CA* activation.

**Will repurposed PI3K cancer therapies be clinically useful as ‘precision’ treatments for PROS?** Recent reports suggest that use of low doses of the p110α-specific inhibitor Alpelisib may yield remarkable clinical improvements in PROS patients, with additional evidence for lesser benefits from mTOR inhibition with Sirolimus. Controlled clinical trials with prespecified end points are urgently needed to confirm these findings and to establish longer-term outcomes.

Box 1 **Clinician’s Corner**

• Tissue growth is regulated by hormones and growth factors. Many of these act on cell surface receptors to trigger activation of intracellular signaling pathways. One major pathway includes the enzyme PI3K. The *PIK3CA* gene encodes a critical component of this enzyme.
• Powerful evidence for the importance of PI3K in growth regulation is the high frequency of activating *PIK3CA* mutations in solid cancers. For this reason, major effort has been invested over 15 years in generating potential cancer drugs to inhibit the abnormally activated enzyme. These drugs, used at high doses, have shown only modest benefits, with high rates of side effects in cancer trials to date.
• More recently, cancer-associated *PIK3CA* mutations have also been shown to cause a wide range of clinical overgrowth disorders, many with different syndromic names based on the pattern of overgrowth. The causal *PIK3CA* mutations almost invariably are not inherited, but instead arise during development and thus are found in only some parts of the body. An asymmetric, patchy distribution of excess growth is a hallmark of this group of syndromes, now suggested to be unified by the label *PIK3CA*-related overgrowth syndrome (PROS). Overgrowths of fat tissue, blood vessels, skin, or the brain are particularly prominent.
• The need for novel medical therapies is large. Major clinical complications in PROS arise from vascular crises and compressive phenomena, and until now the mainstay of therapy has been debulking procedures. Evidence is now emerging that low doses of PI3K inhibitors, initially developed for cancer, may be highly beneficial in PROS, limiting excess soft-tissue growth and potentially reducing need for surgery. Thus, controlled trials are urgently required.
• The study of isolated *PIK3CA* activation in PROS also offers the opportunity to learn more about the fundamental role of PI3K in human development and cancer. In addition to detailed prospective studies of affected patients, their tissues and cells, creation of experimental disease models will be of great importance. Aided by advanced genome editing technologies, animal and stem cell models are now rapidly being developed to help address key unanswered questions.

Alt-text: Box 1

## Glossary

**Carcinoma**: cancer arising from an epithelium.
**CreER**: a tamoxifen-dependent Cre recombinase enzyme commonly used in mouse genetics to generate inducible gene expression or knockout.
**CRISPR-based gene editing**: a powerful approach to genetic engineering, repurposing a bacterial adaptive immune system mechanism. It utilizes a gene-specific guide RNA, a so-called Cas9 nuclease, and often a nucleic acid repair template, either inducing insertion/deletion loss-of-function mutations or knocking in desired mutations.
**Epidermal nevi**: benign localized overgrowth in the epidermis, either present at birth or developing during early childhood.
**Exosomes**: 40–150 nm vesicles arising from endosomal compartments within the cell, and shown to function in local and distant intercellular communication.
**Germline transmission**: the passing of a genetic mutation from parent to offspring via a germ cell (egg or sperm). The mutation is present in every cell of the offspring.
**Induced pluripotent stem cells (iPSCs)**: pluripotent stem cells obtained by direct reprogramming of differentiated cells. These are phenotypically indistinguishable from embryonic stem cells, with the capacity to yield any cell type in the human body.
**Isogenic**: having the same or nearly identical genetic background.
**Mosaicism**: the presence of two or more genetically distinct cell populations in an individual who developed from a single fertilized egg. Genetic mosaicism arises from the acquisition of postzygotic mutations.
**Neural crest**: a transitory neuroectodermal structure formed during embryogenesis. It has the unique property of giving rise to both neural and mesenchymal cell types, which are otherwise strictly derived from either ectodermal or mesodermal germ layers, respectively.
**Neuroectoderm**: derivative of the ectoderm, one of the three embryonic germ layers. Gives rise to neural tissues.
**Oncogene-induced senescence**: a tumor-suppressing mechanism whereby oncogene-induced signaling triggers irreversible growth arrest.
**Postzygotic mutations**: DNA mutations that are not inherited from either parent, arising instead after the single-cell zygote stage of development.
**PTEN hamartoma tumor syndrome**: a group of rare overgrowth disorders caused by heterozygous loss-of-function mutations of phosphatase and tensin homolog (*PTEN*) in the germline.
**Seborrheic keratoses**: benign keratinocyte-derived tumors developing during adulthood and appearing as demarcated brownish plaques predominantly localized in the head, neck, and trunk.
**Self-renewal**: the process wherein cell division leads to regeneration of the original cell type.
**Stemness**: a property of stem cells characterized by a capacity for self-renewal and an undifferentiated state. Often used interchangeably with ‘pluripotency’ in the context of pluripotent stem cells.
