# Supplementary material for: Cancer-Associated PIK3CA Mutations in Overgrowth Disorders
Source: Trends Mol Med. 2018 Oct;24(10):856–70. doi: 10.1016/j.molmed.2018.08.003 (PMC6185869; doi:10.1016/j.molmed.2018.08.003)
Supplement: Supplementary file 1 [file mmc1.pdf]

Supplementary material

Cancer-associated *PIK3CA* mutations in overgrowth disorders

Ralitsa R. Madsen[1,2], Bart Vanhaesebroeck[3], Robert K. Semple[4,1,2,*]

[1]Metabolic Research Laboratories, Wellcome Trust-MRC Institute of Metabolic Science, University of Cambridge, Cambridge CB2 0QQ, UK

[2]The National Institute for Health Research Cambridge Biomedical Research Centre, Cambridge, UK

[3]UCL Cancer Institute, Paul O'Gorman Building, University College London, 72 Huntley Street London WC1E 6DD, UK

[4]Centre for Cardiovascular Sciences, Queens Medical Research Institute, University of Edinburgh, Little France Crescent, Edinburgh EH16 4TJ, UK

* Correspondence: rsemple@ed.ac.uk (Robert K. Semple)

Reference	Activating *PIK3CA* variants studied	Main cell type	Expression method	Main findings specific to *PIK3CA* variants
[1]	E542K, E545K, H1047R	Chicken embryonic fibroblasts	Overexpression	Comparable increase in immunoprecipitated lipid kinase activity across mutants. Mutants promote GF-independent AKT/mTORC1 activation (H1047R more potent) and enhanced PDGF-induced activation. Mutants induce cellular transformation: H1047R somewhat more potent (No wild-type comparator). Favorable response to mTOR inhibition with rapamycin.
[2]	E545K, H1047R	Human colorectal cancer cell lines (HCT116, DLD1)	Homologous recombination to disrupt mutant or wild-type allele, yielding cells with single functional *PIK3CA* gene)	Mutants have increased lipid kinase activity, somewhat more potent for H1047R (recombinant proteins). Mutants promote GF-independent AKT activation, with additional increase in the presence of serum. No differences in growth between wild-type and mutant cells in the presence of optimal serum concentrations, but increased resistance to apoptosis in mutant cells upon serum reduction or TRAIL stimulation. Metastatic tumor formation upon injection of mutant cells into athymic nude mice. Favourable response to pan-PI3K inhibition with LY294002.
[3]	**R38H**, G106V, **C420R**, R453Q, **E542K**, **E545K**, M1043I, **H1047R** (mutations in **bold** only in cellular assays)	Mouse NIH3T3 cells	Overexpression	Immunoprecipitated mutants showed similar increase in lipid kinase activity except R38H which showed only a modest increase. Strong serum-independent AKT activation downstream of C420R, E542K, E545K and H1047R but only modest with R38H. Translated into similar differences in focus-forming ability (R38H similar to wild-type), anchorage-independent growth (modest for R38H) and morphological changes (none observed for R38H, most for C420R). Enhanced mTORC1-dependent signaling.
[4]	E545K, H1047R	Non-transformed human mammary epithelial cells (MCF10A)	Overexpression	Immunoprecipitated mutants showed increased lipid kinase activity. Mutants exhibit enhanced GF-independent as well as GF-dependent activation of AKT, in addition to increased latency for ERK and S6 dephosphorylation upon GF removal. Enhanced proliferation of mutant cells observed only in the absence of GFs. Mutants promote anchorage-independent survival, resistance to chemotherapy (Paclitaxel) as well as altered mammary acinar morphogenesis *in vitro*. Favorable effect of mTOR inhibition with rapamycin or pan-PI3K inhibition with LY294002.
[5]	R38H, K111N, N345K, C420R, P539R, E542K, E545K, E545A, E545G, Q546K, Q546P, H701P, T1025S, M1043I, M1043V, H1047R, H1047L	Chicken embryonic fibroblasts	Overexpression	Increased lipid kinase activity of most immunoprecipitated rare variants, with several of them *on par* with the activity of hotspot mutations. Except H701P and wild-type p110α, all other mutants induced cellular transformation albeit with different efficiencies. Evidence of increased GF-independent AKT/mTORC1 activation downstream of multiple mutants but interpretation confounded by differences in p110α overexpression. Favorable effect of mTOR inhibition with rapamycin.

[6]	R38C, R88Q, R108H, E365K, G1049R, E545K, H1047R	Immortalized human mammary epithelial cells (HMLE)<br>Human osteosarcoma cells (U2OS)<br>Porcine aortic endothelial (PAE) cells	Overexpression	Single Western blot showing that compared to wild-type p110α, overexpression of the different rare mutants in U2OS cells results in enhanced phosphorylation of AKT, GSK3β, FOXO1/3 and S6.<br>Stronger AKT activation by H1047R compared to E545K in U2OS cells.<br>H1047R promotes anchorage independent growth.<br>Evidence that mutant p110α-H1047R cooperates with RAS-G12V to enhance cellular transformation *in vitro*, however control experiments with wild-type p110α are missing.
[7,8]	E542K, E545K, H1047R	[7]: chicken p110α used<br>[8]: human p110α used	Overexpression	Helical and kinase domain mutations act synergistically in cell transformation, corresponding to stronger GF-independent activation of AKT and S6K. Upon single overexpression, H1047R more potent than E542K/E545K, and E542K more potent than E545K.<br>Complete abrogation of cellular transformation by p110α-H1047R that is unable to bind p85, despite constitutive AKT/S6K activation – at least in studies with chicken p110α.<br>Deficiency in RAS binding impairs the transforming ability of helical domain mutants, but not that of p110α-H1047R despite a substantial decrease in AKT activation.
[9]	E542K, E545A, H1047R	IL-3 dependent murine pro-B cell (Ba/F3)	Overexpression	Mutant p110α variants, but not wild-type p110α, enable IL-3-independent growth. Subsequent phenotyping studies performed without wild-type control, although Myr-p110α included as positive control. These studies suggest enhanced AKT-dependent, but mTORC1-independent, survival in mutant cells. Rapid expansion and tumorigenic spread of cells expressing p110α-E545A in allografted mice. Favorable response to *in vitro* pan-PI3K inhibition with LY294002 or AKT inhibition with Akt IV.
[10]	E545K, H1047R	Non-transformed human mammary epithelial cells (MCF10A)	Endogenous (heterozygous knock-in)	Endogenous mutant expression does not promote cellular transformation (no soft agar colony formation or tumor formation in nude mice). Mutants promote proliferation with and without EGF in the medium. EGF-independent phosphorylation of AKT and GSK3β, but not mTORC1 (S6K1), in mutant cells. Accompanied by increased Cyclin D1 levels, with no difference in p21/p27 phosphorylation. Mutant cells have a lower threshold for EGF-induced ERK and mTORC1 activation, and further demonstrate enhanced AKT activation in response to EGF. Sensitivity to mTOR inhibition with rapamycin only observed in EGF-cultured mutant cells. Favorable response to pan-PI3K inhibition with wortmannin or LY294002, as well as MEK inhibition with U0126. Favorable response to GSK3 inhibition *in vitro* and *in vivo* (xenograft assay using mutant HCT116 from Samuels *et al.* 2005).
[11]	E545K, H1047R	Human breast cancer cell line (MDA-MB-231)	Knockdown of endogenous p110α and lentiviral replacement with bovine p110α (wild-type or mutant)	GF-independent activation of AKT in mutant cells. Enhanced growth of mutant cells compared to wild-types, most notable in the absence of GF. Enhanced migration of mutant cells; increased directional chemotaxis of cells expressing E545K. Enhanced growth of xenograft mouse tumors derived from mutant cells. Both mutants lead to metastatic activity, but evidence that this is increased in cells expressing E545K.

[12]	E545K, H1047R	Non-transformed human mammary epithelial cells (MCF10A) with *HER2 (ERBB2)* overexpression	Overexpression	H1047R and E545K enhance the growth and migration of *HER2*-overexpressing cells; H1047R more potent. In GF-replete conditions, no difference in proliferation compared to HER2/WT cells, but enhanced proliferation of HER2/E545K and HER2/H1047R cells observed upon serum removal.<br>In GF-replete conditions, elevated AKT/mTORC1 activation observed in HER2/H1047R cells but not HER2/E545K cells. GF-independent AKT activation observed in both HER2/E545K and HER2/H1047R cells. Different mechanisms identified whereby E545K and H1047R enhance HER2-mediated transformation. Favorable response to p110α-specific inhibition with BEZ235, pan-PI3K inhibition with LY294002 and combined inhibition of p110α/HER2 with BEZ235/lapatinib.
[13]	R93W, R93Q, G106V, G160R, K111E, V344A, G364R, E365K, delP449_L455, E453A, E453K, H1047R	Human osteosarcoma cells (U2OS)	Overexpression	Limited functional phenotyping by a single Western blot, with evidence of variable GF-independent AKT hyperactivation downstream of several variants; confounded by variable expression levels of p110α.
[14]	R88Q, K111N, N345K, C420R, P539R, E542K, E545K, E545A, E545G, Q546K, M1043I, H1047L, H1047R, G1049R	Human embryonic kidney cells (HEK293)<br>Human colorectal cancer cells (DLD1)	Overexpression (HEK293)<br>Endogenous (DLD1: homologous recombination used to disrupt either the mutant or wild-type allele, resulting in cells with a single functional *PIK3CA* gene)	Immunoprecipitation studies in HEK239 cells overexpressing the listed variants along with IRS1, demonstrating GF-independent association with IRS1 and the following mutants: K111N, E542K, E545K, E545G, Q546K, M1043I.<br>Detailed studies of the interaction between E545K and IRS1, showing that it occurs independent of p85 binding and IRS1 tyrosine phosphorylation. This E545K-IRS1 interaction was shown to be important for the growth of xenograft tumors derived from mutant cells; such growth can be inhibited with a competing, recombinant p110α-E545K mutant peptide.
[15]	E545K, H1047R	Non-transformed human mammary epithelial cells (MCF10A)	Endogenous (heterozygous knock-in)	Only a small part of the study looks at class IA PI3K signaling in *PIK3CA* mutant-expressing MCF10As. Single Western blot showing increased AKT activation in starved mutant cells; possibly stronger with H1047R compared to E545K. In comparison, the EGF signaling response is similar in both mutant cell lines and the parental cell line. Both mutant cell lines show increased migration at baseline and in response to EGF stimulation; reversal upon pan-PI3K inhibition with either PI103 or LY294002.

[16]	E545K, H1047R (+/- *KRAS*-G12V)	Non-transformed human mammary epithelial cells (MCF10A)	Endogenous / Overexpression	Study examines cooperativity between *PIK3CA* hotspot mutations and *KRAS*-G12V. Evidence for synergistic oncogenic effects both *in vitro* and xenograft assays *in vivo*. Minimal increase in both S6K1 and RSK1 phosphorylation with single *PIK3CA* hotspot mutants, but substantial increase in cells with *PIK3CA* and *KRAS* mutations, each knocked in heterozygously (assessed in the absence of EGF). Double-mutant cells also have increased phosphorylation of S6, but not 4E-BP1. Increased ERK phosphorylation in double-mutant cells, but slightly decreased AKT phosphorylation compared to cells with mutant *PIK3CA* only. The high ERK phosphorylation in *PIK3CA* mutant cells is not dependent on RAS binding, but AKT phosphorylation in both mutants appears to be (modest decrease when p110α RBD mutated and only single Western blots shown; these particular results obtained in the context of overexpression).
[17]	P447_L455del (447del), H450_P458del (450del)	Non-transformed human mammary epithelial cells (MCF10A)	Overexpression	Computational modeling and biochemical studies suggesting that both deletions destabilise the interaction between p85 and p110α. More unstable p110α protein is still able to induce hyperactivation of PI3K/AKT/mTORC1 signaling and modestly increase in ERK1/2 phosphorylation, GF-independent growth and cellular transformation. Treatment with the p110α-specific inhibitor BYL719 leads to phenotypic reversal. MCF10A cells with endogenous expression of either E545K or H1047R used as controls in some of the cellular studies, with evidence that E545K-expressing MCF10A cells can survive better than cells expressing H1047R in the absence of GFs (insulin and EGF).
[18]	E545K, H1047R	Human umbilical vein endothelial cells (HUVEC)	Overexpression	Both mutants promote an increase in cell size, proliferation and senescence with increased cell size (20 % of mutant cells). Some phenotypes enhanced further by GF stimulation. Mutant cell lines exhibit increased sprouting angiogenesis at baseline and in response to VEGFA. Migratory index comparable to control cells overexpressing wild-type p110α. Enhanced GF-independent AKT phosphorylation but minimal effect on mTORC1-dependent target phosphorylation. GF stimulation does not further increase pathway activation in mutant-expressing cells. Favorable response to pathway inhibition with BEZ235 (dual PI3K/mTOR), everolimus (mTOR) and MK2206 (AKT), with best response following BEZ235 treatment.
[19]	R88Q, C90Y, E542K, E545K, H1047R, M1043V (+/- *HRAS*-G12V)	Immortalized human astrocytes	Overexpression	All comparisons are to cells overexpressing wild-type *PIK3CA*. In the absence of *HRAS*-G12V overexpression, GF-independent AKT phosphorylation increased with both helical and kinase domain mutants but not ABD mutants (R88Q, C90Y). With *HRAS*-G12V, GF-independent AKT phosphorylation is observed in all mutants except C90Y, but the effect of R88Q is only slightly higher compared to overexpression of wild-type *PIK3CA*. No effect of mutant *PIK3CA* on ERK phosphorylation regardless of *HRAS* status. In the presence of serum, only slightly increased proliferation in all *PIK3CA* mutants except C90Y; no longer observed in the presence of *HRAS*-G12V which causes aggressive behavior on its own. A modest increase in migration *in vitro* with helical and kinase domain mutants both with and without *HRAS*-G12V; also observed for R88Q but only in the presence of *HRAS*-G12V. Increased colony formation only observed with H1047R (trend towards an increase with E542K); colony formation is increased with *HRAS*-G12V, and there is no further potentiation with H1047R. E542K and H1047R potentiate tumorigenesis of *HRAS*-G12V-expressing cells in xenograft assays *in vivo*; minimal effect of R88Q in this setting, and C90Y not tested. Some evidence that synergy between pan-PI3K inhibition (BKM120) and MEK inhibition (selumetinib) is determined both by the *PIK3CA* and *HRAS* genotype.

S1: Cellular studies examining multiple activating *PIK3CA* mutations across a range of cells and expression strategies. ABD: adaptor-binding domain; EGF: epidermal growth factor; IL-3: interleukin-3; GF: growth factor; HER2, epidermal growth factor receptor 2; Myr-p110α: myristoylated p110α; PDGF: platelet-derived growth factor; TRAIL: TNF-related apoptosis-inducing ligand. This table includes studies that have compared multiple *PIK3CA* variants to assess the available evidence for mutant-specific phenotypes; studies examining single *PIK3CA* variants in isolation are excluded. Note that this table does not cover results from high-throughput proteomics/transcriptomics studies (for these, see [20–27]).

Drug	Company	Mechanism	Specificity[a]	Status	Conditions
BKM120 (Buparlisib)	Novartis	ATP-competitive	**Pan-PI3K:** IC50 for p110α/β/δ/γ in cell-free assays 52/166/116/262 nM, respectively.	Phase II	Metastatic breast cancer, triple-negative breast cancer, NSCLC, endometrial cancer, thyroid cancer, esophageal cancer, recurrent/progressive head and neck cancer, recurrent glioblastoma, high-risk prostate cancer, diffuse large B-cell lymphoma, mantle cell lymphoma, follicular lymphoma, metastatic urothelial carcinoma, relapsed/refractory thymomas, recurrent/refractory PCNSL/SCNSL
BAY 80-6946 (Copanlisib)	Bayer	ATP-competitive	**Pan-PI3K:** IC50 for p110α/β/γ/δ in cell-free assays 0.5/3.7/6.4/0.7 nM, respectively.	Phase II	Non-Hodgkin's lymphoma, persistent/recurrent endometrial cancer
GDC-0032 (Taselisib)[b]	Roche	ATP-competitive	**PI3K β-sparing:** targets p110α/δ/γ with Ki 0.29/0.12/0.97nM respectively, and >10 fold selective over p110β.	Phase I/II, II	PROS, recurrent stage IV SCLC, refractory solid tumors/lymphomas/multiple myeloma
XL147 (SAR245408, Pilaralisib)	Sanofi	ATP-competitive	**PI3K β-sparing:** IC50 for p110α/δ/γ in cell-free assays 39/36/23 nM, respectively. Less potent to p110β.	Phase I/II	Advanced/recurrent endometrial carcinoma
BYL719 (Alpelisib)	Novartis	ATP-competitive	**p110α:** IC50 5 nM in cell-free assay, with minimal effect on p110β/γ/δ.	Phase II	PROS, Metastatic breast cancer, recurrent/metastatic head and neck cancer, esophageal cancer, SCLC, lung adenocarcinoma
BGT226 (NVP-BGT226)	Novartis	ATP-competitive	**δ-sparing PI3K/mTOR:** IC50 for p110α/β/γ in cell-free assays 4/63/38 nM, respectively.	Phase II	Advanced solid tumors, advanced breast cancer, PHTS
LY3023414	Eli Lilly	ATP-competitive	**PI3K/mTOR**: inhibitor of all class I PI3K isoforms, mTOR and DNA-PK.	Phase II	Pediatric patients with relapsed or refractory advanced solid tumors, non-Hodgkin's lymphoma, or histiocytic disorders
BEZ235 (NVP-BEZ235, Dactolisib)	Novartis	ATP-competitive	**PI3K/mTOR**: IC50 4/5/7/75 nM for p110α/γ/δ/β, respectively, and 6 nM for mTOR (P70S6K); cell-free assays. IC50 of 21 nM for ATR in 3T3TopBP1-ER cells	Phase II	Urothelial carcinoma
PQR309	PIQUR Therapeutics	ATP-competitive	**PI3K/mTOR**	Phase I/II, II	Relapsed/refractory lymphoma, lymphoma, glioblastoma
PF-05212384 (Gedatolisib, PKI-587)	Pfizer	ATP-competitive	**PI3K/mTOR:** potent dual inhibitor of p110α/γ and mTOR with IC50 of 0.4/5.4 nM and 1.6 nM, respectively, in cell-free assays.	Phase II	AML, myelodysplastic syndrome, endometrial cancers
PF-04691502	Pfizer	ATP-competitive	**PI3K/mTOR:** p110α/β/δ/γ and mTOR dual inhibitor with Ki of 1.8 nM/2.1 nM/1.6 nM/1.9 nM and 16 nM in cell-free assays, little activity against either	Phase II	Endometrial cancers

			VPS34, AKT, PDK1, P70S6K1, MEK, ERK, p38, or JNK.		
SF1126	SignalRx	ATP competitive	**PI3K/mTOR**	Phase II	Metastatic squamous neck cancer with occult primary squamous cell carcinoma
AZD5363	AstraZeneca	ATP-competitive	**AKT:** IC50 of 3/8/8 nM for AKT1/2/3 respectively in cell-free assays.	Phase II	Refractory solid tumors/lymphomas/multiple myeloma
ARQ-092 (Miransertib)	ArQule	ATP-competitive	**AKT**	Phase I/II	Proteus Syndrome, PROS
TAS-117	Taiho Pharmaceutical	Allosteric	**AKT**	Phase II	Solid tumors
MK-2206	Merck	Allosteric	**AKT:** IC50 for AKT1/2/3 in cell-free assays 8/12/65 nM, respectively; no inhibitory activities observed against 250 other protein kinases.	Phase II	Metastatic colorectal cancer, recurrent/advanced endometrial cancer, advanced breast cancer, NSCLC, SCLC, thymic malignancies
TAK-228 (MLN0128, INK128)	Takeda Pharmaceutical	ATP-competitive	**mTOR:** IC50 of 1 nM for mTOR in cell-free assays; >200-fold less potent to class I PI3K isoforms.	Phase II	Soft tissue sarcoma
Rapamycin (Sirolimus) and rapalogs (CCI-779/Temsirolimus, RAD001/Everolimus)	Multiple	Allosteric	**mTOR:** rapamycin (Sirolimus) is a specific mTOR inhibitor with IC50 of 0.1 nM in HEK293 cells.	Phase I/II, II, III, IV	Low-grade glioma, advanced/refractory tumors, solid tumors, pancreatic cancer, metastatic breast cancer, pheochromocytoma, extra-adrenal paraganglioma, non-functioning carcinoid, Kaposi's sarcoma, NSCLC, advanced renal cell carcinoma, PROS, vascular malformations

S2: PI3K pathway inhibitors in clinical trials (phase II+) as monotherapy. These results were collated on 30/10/2017 from https://clinicaltrials.gov, selecting class IA PI3K pathway inhibitors in clinical trials (phase II+) as monotherapy. Trials were only included if classified as completed/terminated/suspended or currently recruiting/ongoing. [a]If available, specificity information taken from: http://www.selleckchem.com; IC50 is the half-maximal inhibitory concentration (assay-dependent), and Ki the biochemical inhibitor binding constant (intrinsic). [b]In June 2018, Roche informed that they will discontinue Taselisib (GDC-0032) based on modest effects and high toxicity in a breast cancer clinical trial of the drug in combination with Fulvestrant (Trial no.: NCT02340221). AML: Acute Myeloid Leukemia; NSCLC: Non-Small Cell Lung Cancer; PCNSL: Primary Central Nervous System Lymphoma; PROS: *PIK3CA*-Related Overgrowth Spectrum; PHTS: PTEN Hamartoma Tumor Syndrome; SCLC: squamous cell lung cancer; SCNSL: Secondary Central Nervous System Lymphoma.

References

1 Kang, S. et al. (2005) Phosphatidylinositol 3-kinase mutations identified in human cancer are oncogenic. Proc. Natl. Acad. Sci. **102**, 802–807.
2 Samuels, Y. et al. (2005) Mutant PIK3CA promotes cell growth and invasion of human cancer cells. Cancer Cell **7**, 561–573.
3 Ikenoue, T. et al. (2005) Functional analysis of PIK3CA gene mutations in human colorectal cancer. Cancer Res. **65**, 4562–4567.
4 Isakoff, S. J. et al. (2005) Breast cancer-associated PIK3CA mutations are oncogenic in mammary epithelial cells. Cancer Res. **65**, 10992–11000.
5 Gymnopoulos, M. et al. (2007) Rare cancer-specific mutations in PIK3CA show gain of function. Proc. Natl. Acad. Sci. **104**, 5569–5574.
6 Oda, K. et al. (2008) PIK3CA cooperates with other phosphatidylinositol 3′-kinase pathway mutations to effect oncogenic transformation. Cancer Res. **68**, 8127–8136.
7 Zhao, L. and Vogt, P. K. (2008) Helical domain and kinase domain mutations in p110alpha of phosphatidylinositol 3-kinase induce gain of function by different mechanisms. Proc. Natl. Acad. Sci. U. S. A. **105**, 2652–2657.
8 Zhao, L. and Vogt, P. K. (2010) Hot-spot mutations in p110α of phosphatidylinositol 3-kinase (PI3K): Differential interactions with the regulatory subunit p85 and with RAS. Cell Cycle **9**, 596–600.
9 Horn, S. et al. (2008) Mutations in the catalytic subunit of class IA PI3K confer leukemogenic potential to hematopoietic cells. Oncogene **27**, 4096–106.
10 Gustin, J. P. et al. (2009) Knockin of mutant PIK3CA activates multiple oncogenic pathways. Proc. Natl. Acad. Sci. **106**, 2835–40.
11 Pang, H. et al. (2009) Differential enhancement of breast cancer cell motility and metastasis by helical and kinase domain mutations of class IA phosphoinositide 3-kinase. Cancer Res. **69**, 8868–8876.
12 Chakrabarty, A. et al. (2010) H1047R phosphatidylinositol 3-kinase mutant enhances HER2-mediated transformation by heregulin production and activation of HER3. Oncogene, Nature Publishing Group **29**, 5193–203.
13 Rudd, M. L. et al. (2011) A unique spectrum of somatic PIK3CA (p110α) mutations within primary endometrial carcinomas. Clin. Cancer Res. **17**, 1331–1340.
14 Hao, Y. et al. (2013) Gain of Interaction with IRS1 by p110a-Helical Domain Mutants Is Crucial for Their Oncogenic Functions. Cancer Cell, Elsevier Inc. **23**, 583–593.
15 Juvin, V. et al. (2013) Signaling via Class IA Phosphoinositide 3-Kinases (PI3K) in Human, Breast-Derived Cell Lines. PLoS One **8**.
16 Wang, G. M. et al. (2013) Single copies of mutant KRAS and mutant PIK3CA Cooperate in immortalized human epithelial cells to induce tumor formation. Cancer Res. **73**, 3248–3261.
17 Croessmann, S. et al. (2018) PIK3CA C2 Domain Deletions Hyperactivate Phosphoinositide 3-kinase (PI3K), Generate Oncogene Dependence, and Are Exquisitely Sensitive to PI3K α Inhibitors. Clin. Cancer Res. **24**, 1426–1435.
18 di Blasio, L. et al. (2018) PI3K/mTOR inhibition promotes the regression of experimental vascular malformations driven by PIK3CA-activating mutations. Cell Death Dis. **9**, 45.
19 Miller, C. R. et al. (2018) PIK3CA missense mutations promote glioblastoma pathogenesis , but do not enhance targeted PI3K inhibition. PLoS One 1–21.
20 Wu, X. et al. (2014) Activation of diverse signalling pathways by oncogenic PIK3CA mutations. Nat. Commun. **5**.
21 Hart, J. R. et al. (2015) The butterfly effect in cancer: A single base mutation can remodel the cell. Proc. Natl. Acad. Sci. **112**, 1131–1136.
22 Blair, B. G. et al. (2015) A phosphoproteomic screen demonstrates differential dependence on HER3 for MAP kinase pathway activation by distinct PIK3CA mutations. Proteomics **15**, 318–326.
23 Zahari, M. S. et al. (2015) Activating mutations in PIK3CA lead to widespread modulation of the tyrosine phosphoproteome. J. Proteome Res. **14**, 3882–3891.
24 Kiselev, V. Y. et al. (2015) Perturbations of PIP3 signalling trigger a global remodelling of mRNA landscape and reveal a transcriptional feedback loop. Nucleic Acids Res. gkv1015.
25 Dogruluk, T. et al. (2015) Identification of Variant-Specific Functions of PIK3CA by Rapid Phenotyping of Rare Mutations. Cancer Res. **75**, 5341–5354.
26 Moniz, L. S. et al. (2017) Phosphoproteomic comparison of Pik3ca and Pten signalling identifies the nucleotidase NT5C as a novel AKT substrate. Sci. Rep. **7**, 39985.
27 Zhang, Y. et al. (2017) A Pan-Cancer Proteogenomic Atlas of PI3K/AKT/mTOR Pathway Alterations. Cancer Cell 1–13.
